# Selection of marine bacterial consortia efficient at degrading chitin leads to the discovery of new potential chitin degraders

**DOI:** 10.1128/spectrum.00886-24

**Published:** 2024-09-24

**Authors:** Laurence Meunier, Rodrigo Costa, Tina Keller-Costa, David Cannella, Etienne Dechamps, Isabelle F. George

**Affiliations:** 1 Laboratory of Ecology of Aquatic Systems, Brussels Bioengineering School, Université Libre de Bruxelles (ULB), Brussels, Belgium; 2 Institute for Bioengineering and Biosciences (iBB) and Institute for Health and Bioeconomy (i4HB), Instituto Superior Técnico (IST), Universidade de Lisboa, Lisbon, Portugal; 3 Department of Bioengineering, Instituto Superior Técnico (IST), Universidade de Lisboa, Lisbon, Portugal; 4 PhotoBioCatalysis Unit, Crop Nutrition and Biostimulation Lab (CPBL) and Biomass Transformation Lab (BTL), Brussels Bioengineering School, Université Libre de Bruxelles, Brussels, Belgium; Panepistemio Thessalias Tmema Geoponias Ichthyologias kai Ydatinou Periballontos, Volos, Greece

**Keywords:** microbial communities, enrichment cultures, chitinase, marine sponge, size exclusion chromatography

## Abstract

Chitin degradation is a keystone process in the oceans, mediated by marine microorganisms with the help of several enzymes, mostly chitinases. Sediment, seawater, and filter-feeding marine invertebrates, such as sponges, are known to harbor chitin-degrading bacteria and are presumably hotspots for chitin turnover. Here, we employed an artificial selection process involving enrichment cultures derived from microbial communities associated with the marine sponge *Hymeniacidon perlevis*, its surrounding seawater and sediment, to select bacterial consortia capable of degrading raw chitin. Throughout the artificial selection process, chitin degradation rates and the taxonomic composition of the four successive enrichment cultures were followed. To the best of our knowledge, chitin degradation was characterized for the first time using size exclusion chromatography, which revealed significant shifts in the numbered average chitin molecular weight, strongly suggesting the involvement of endo-chitinases in the breakdown of the chitin polymer during the enrichment process. Concomitantly with chitin degradation, the enrichment cultures exhibited a decrease in alpha diversity compared with the environmental samples. Notably, some of the dominant taxa in the enriched communities, such as *Motilimonas*, *Arcobacter*, and *Halarcobacter*, were previously unknown to be involved in chitin degradation. In particular, the analysis of published genomes of these genera suggests a pivotal role of *Motilimonas* in the hydrolytic cleavage of chitin. This study provides context to the microbiome of the marine sponge *Hymeniacidon perlevis* in light of its environmental surroundings and opens new ground to the future discovery and characterization of novel enzymes of marine origin involved in chitin degradation processes.

**IMPORTANCE:**

Chitin is the second most abundant biopolymer on Earth after cellulose, and the most abundant in the marine environment. At present, industrial processes for the conversion of seafood waste into chitin, chitosan, and chitooligosaccharide (COS) rely on the use of high amounts of concentrated acids or strong alkali at high temperature. Developing bio-based methods to transform available chitin into valuable compounds, such as chitosan and COS, holds promise in promoting a more sustainable, circular bioeconomy. By employing an artificial selection procedure based on chitin as a sole C and N source, we discovered microorganisms so-far unknown to metabolize chitin in the rare microbial biosphere of several marine biotopes. This finding represents a first important step on the path towards characterizing and exploiting potentially novel enzymes of marine origin with biotechnological interest, since products of chitin degradation may find applications across several sectors, such as agriculture, pharmacy, and waste management.

## INTRODUCTION

Chitin is a polymer of N-acetyl-glucosamine (GlcNAc) linked *via* β-1,4-glycosidic bonds. It is the second most abundant biopolymer on Earth after cellulose, and the most abundant in the marine environment where it is an important source of organic carbon and nitrogen (1, 2). It is present in many organisms, such as crustaceans, insects, most fungi, and some algae, serving as a structural component (3, 4). Every year, approximately 10^11^ T of chitin are produced by living organisms in aquatic ecosystems (5, 6), and more than 10,000 T can be extracted from shellfish waste provided by the seafood industry (7). At present, industrial processes for the conversion of seafood waste into chitin, chitosan, and chitooligosaccharide (COS) rely on the use of high amounts of concentrated acids or strong alkali at high temperature (8–10). Developing bio-based methods to transform such available chitin into valuable compounds, such as chitosan and COS holds promise in promoting a more sustainable, circular bioeconomy.

Although chitin is difficult to decompose due to its crystalline structure (9), chitinolytic bacteria can degrade chitin through the action of chitinases. The chitinolytic pathways have been thoroughly described for a limited number of bacterial taxa, such as the marine bacteria *Vibrio* spp. (e.g., *Vibrio furnisii, Vibrio cholerae, Vibrio harveyi,* and *Vibrio carchariae*) (11–15) and the soil bacterium *Serratia marcescens* (16). Recently, the screening of 30 microbial metagenomes of seawater, sediment, octocoral, and sponge samples for genes encoding enzymes involved in chitin degradation and their taxonomical assignment revealed great richness among chitinolytic communities from those biotopes (17). In addition, the putative role of uncultivated *Gammaproteobacteria* and *Chloroflexi* symbionts in chitin processing was unveiled. Similarly, the analysis of 66 genomes assembled from the metagenome of octocoral species and seawater showed for the first time the presence of genes associated with chitin degradation in *Endozoicomonadaceae* and chitin deacetylation in *Metamycoplasmataceae* and Ca. *Thioglobaceae* (18). These studies highlight that the census of marine chitinolytic bacteria and their enzymatic arsenal, so far largely based on pure cultures, is far from complete. Chitinases are glycosyl hydrolases (GHs) that hydrolyze the β-1,4-glycosidic bonds between the GlcNAc residues to produce soluble COS, dimers, and monomers. Two types of chitinases cleave the chitin fibers: endochitinases (EC 3.2.1.14), which are extracellular enzymes that cleave chitin at internal sites and generate multimers of GlcNAc, and exochitinases (EC 3.2.1.52), which cleave progressively chitin polymers, oligomers, and dimers to produce chitobiose (dimer: GlcNAc_2_) and GlcNAc. These resulting chitin degradation products can be taken up by the cells and metabolized into acetate, fructose-6P, and NH_3_ (15). In addition, oxidative enzymes able to degrade chitin have been discovered: the lytic polysaccharide monooxygenase*s* (LPMO, EC 1.14.99.53), today classified as auxiliary activity 10 enzymes (AA10) (19). Their activity produces new chain termini on the chitin structure then aiding the hydrolytic activity of GHs. In contrast, the role of some carbohydrate binding module proteins (CBPs), either complexed to GH or alone, has not been fully elucidated in the context of chitin degradation, although they are present in the chitinolytic machinery of several organisms (20). Finally, chitin can also be deacetylated into chitosan by chitin deacetylases (EC 3.4.1.41), a process presumably more important in soil and sediment than in the water environment (3, 21).

Not all chitinolytic bacteria have the ability to degrade raw chitin fibers; therefore cross-feeding mechanisms frequently occur between “chitin degraders” that act on the chitin insoluble polymer through the action of endochitinases or deacetylases and “chitin utilizers” that consume chitin degradation products, such as COS, GlcNAc, and chitosan (22). Such cross-feeding mechanisms can enhance the activity of chitin degraders (depending on the identity of the cross-feeders) (23). This could be attributed to the consumption of the chitin degradation products, which drives the reaction forwards, and by the removal of metabolites that inhibit the growth of chitin degraders, as it was shown for other cross-feeding mechanisms (24–26). In addition, metabolic by-products excreted by the degraders and utilizers, such as acetate or amino acids, can benefit other organisms that are not involved in chitin degradation (23).

Although chitin is constantly sinking in the ocean in the form of “marine snow” and is present in benthic crustaceans (4), it does not accumulate on the seafloor, and marine sediments contain only traces of chitin (27–29). Indeed, microbial communities in marine sediments hold highly active chitinolytic bacteria that ensure the constant and rapid chitin turnover (30, 31). Chitinolytic bacteria are autochthonous in marine waters as well (3), where they can be found attached to chitinous exoskeletons or particulate detritus in the seawater column (32), or in symbiosis with macroeukaryotic hosts (17, 18). Among the latter, marine sponges have the ability to filter thousands of liters of water per kilogram (wet weight) per day (33), suggesting that they could be favorable biotopes for chitin degraders.

The main objectives of this study were (i) to produce, through an artificial selection process (i.e., enrichment cultures using insoluble chitin as the sole source of carbon and nitrogen), marine multispecies bacterial consortia in the laboratory that are efficient at degrading chitin without trying to single out and purify cultures from the environmental samples the traditional way and (ii) to compare the chitinolytic activity and composition of these bacterial consortia enriched from several marine biotopes. To this end, three natural marine biotopes expected to harbor distinct chitin-degrading communities — namely seawater, sediments, and marine sponges — were used as starting material for the artificial selection experiments. The chosen model host organism was the marine sponge *Hymeniacidon perlevis* (*Demospongiae*, *Halichondrida*), a most likely low microbial abundance (LMA) (34) encrusting sponge commonly found along the European coasts of the Channel, the Northeast Atlantic Ocean, and the Mediterranean Sea (35, 36). The degradation of insoluble chitin in the enrichment cultures was assessed by measuring changes in the molecular weight of the chitin polymer using size exclusion chromatography (SEC) instead of relying on commercial kits that measure a potential degradation activity of short COS. In parallel, the bacterial communities were thoroughly characterized by 16S rRNA gene sequencing.

## MATERIALS AND METHODS

### Sampling

*H. perlevis* specimens (three biological replicates: SP1, SP2, and SP3) and its surrounding seawater and sediment (three biological replicates each : SW1, SW2, and SW3 for seawater and SD1, SD2, and SD3 for sediment) were collected at the beach of Audresselles, France (Lat. 50° 49′14.888N, Long. 1° 35′ 34.354E) at low tide on 6 June 2020. Sponge specimens (about 5 g each), located on the inferior part of rocks in the intertidal zone, were excised with a sterile scalpel and placed individually with surrounding seawater into Ziploc^R^ bags. Surface sediment (maximum 1 cm depth) was sampled with a sterile spoon (c. 2 g/ replicate) and kept in sterile pots. Finally, seawater samples (c. 2 L/ replicate) were stored in sterile bottles. Samples were transported to the laboratory in a cooling box.

### Sample processing

In a sterile hood, sponges were handled with sterilized tweezers to remove macroscopic epibionts and extracellular endobionts such as mussels, gastropods, worms, and algae. Afterward, sponge specimens were cleaned with sterile artificial seawater (ASW) (33.3 g/L of sea salt [Instant Ocean] in MilliQ water). After washing, 0.25 g of each specimen was stored at −80°C for future DNA extraction. Seawater samples (c. 400 mL) were filtered through 0.8-µm nitrocellulose membranes (Whatman, England). Seawater filters and sediment samples (0.25 g/replicate) were stored at −80°C for future DNA extraction.

Microbial cells were enriched from sponge tissue and sediment according to the method described by Esteves *et al.* (37) with minor modifications. To obtain microbial cell enrichments, sponge specimens (2 g each) were crushed using a mortar and a pestle, and sediment replicates (1 g each) were mixed with 50 mL and 9 mL of ASW, respectively, under a sterile hood. The homogenates were then centrifuged once at 4°C at 100×*g* for 15 min to remove large aggregates; the supernatant was then centrifuged twice at 4°C at 300×*g* for 15 min. Afterward, the supernatant was filtered twice on a 10-µm pore size membrane (Whatman, England) and once on a 3-µm pore size membrane (Whatman, England) using a vacuum pump. Finally, the flow through was centrifuged for 20 min at 4°C and 5,200×*g*, and the microbial cell pellet was recovered. To prepare microbial cell enrichments from seawater samples, approximately 400 mL of seawater was filtered on 0.8-µm pore size membranes (Whatman, England) to recover bacteria associated with microeukaryotes (diatoms, microalgae). The membranes were cut into small pieces with a sterile scalpel inside a sterile hood and mixed with 10 mL of half-strength marine broth (ROTH, Navarra, Spain) made with 1:1 v/v MilliQ water: ASW (Instant Ocean). After 2 days of incubation at 20°C and 175 rpm, 2 mL of the culture was centrifuged at 4°C at 5,200×*g* for 20 min and the microbial cell pellet was recovered. Each cell pellet was resuspended in 830 µL of ASW, then mixed with 150 µL of glycerol and 20 µL of pure DMSO. These glycerol stocks were stored at −80°C.

### Artificial selection procedure

Microbial cell suspensions from the three biotopes (three biological replicates each) were used as the starting material for artificial selection of microbial communities through successive transfers of enrichment cultures in a chitin-containing culture medium. In total, nine artificial selection experiments were initiated in this study.

**Fig 1:**
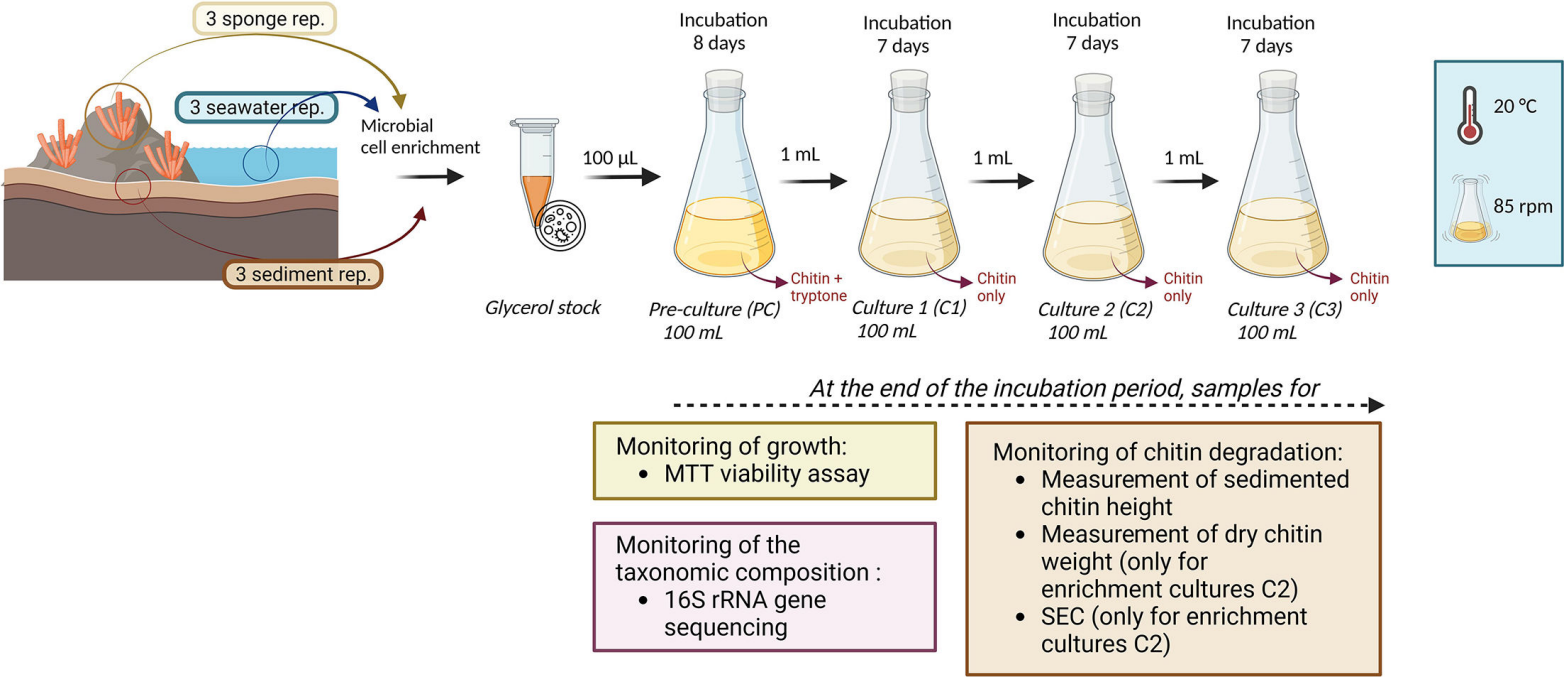
Experimental design of the artificial selection process. Image created with BioRender.com

The artificial selection process consisted of one pre-culture (referred to as “enrichment culture PC”) and three successive cultures (so-called “enrichment cultures C1, C2, and C3”). Briefly, 100 µL of each glycerol stock was inoculated into 100 mL of pre-culture medium at 20°C and 85 rpm. After 8 days of incubation, 1 mL of this PC was added to 100 mL of culture 1 medium. After 7 days of incubation, 1 mL of enrichment culture C1 was transferred to 100 mL of culture 2 medium. This step was repeated once more to generate enrichment culture C3 (Fig. 1).

The culture medium for the preparation of the enrichment cultures (C1, C2, and C3) was composed of 100 mL of autoclaved ASW (Instant Ocean); 0.15 g of KH_2_PO_4_; 1 g of chitin powder extracted from shrimp shells (C7170 from Sigma-Aldrich/Merck, Germany), and 160 µL of a solution of trace elements (for details, see Table S1). The same medium was amended with 0.01 g of tryptone in the preparation of the pre-culture medium (PC) to boost the growth of bacteria at the beginning of the process (Fig. 1).

After each incubation period, the viability of the enrichment cultures was directly assessed using the MTT cell viability assay (described in the supplemental methods). In addition, 5 mL samples were collected for the following analyses: (i) semi-quantitative assessment of chitin degradation as described below, (ii) qualitative characterization of the chitin degradation products by size exclusion chromatography (SEC) after storage of the samples at −80°C, and (iii) DNA extraction after centrifugation (10,000×*g* for 10 min) and storage of the pellet at −20°C.

### Chitin degradation assessment

Chitin degradation was first assessed in all samples in a semi-quantitative way, by recording the height of chitin remaining in the enrichment cultures and settled for 30 min in fully filled 5-mL microtubes (Eppendorf, Germany) (three technical replicates for each measurement). Chitin height was also measured on the negative control sample (in triplicate). The chitin heights (with error bars) over the enrichment cultures were represented in scatter plots using the *ggplot* function from the dplyr package (38) in R.

We applied two additional methodologies to assess chitin degradation in enrichment cultures C2, where chitin was usually observed to be effectively degraded according to chitin height measurements. First, the weight of chitin remaining in the enrichment cultures was measured. For that purpose, 5 mL of the liquid culture (in triplicate) was centrifuged at 2,000×*g* for 5 min, and the pellet was washed twice with MilliQ water. The chitin pellet was then dried at 70°C in heating blocks (DRB200; Hach, USA) until reaching a constant weight and weighted. Second, size exclusion chromatography (SEC) was used to determine the molecular mass parameters of the chitin polymers: numbered average molecular weight (Mn), weight average molecular weight (Mw), and polydispersity (PDI), with three technical replicates conducted for each sample. The molecular mass parameters of the chitin polymers were also determined for the negative control (C-; in triplicate). The SEC protocol was applied to dry chitin pellets obtained as described previously; this protocol is described in the supplemental material.

The polymeric parameters (Mn, Mw, and PDI) can be calculated for any region of a SEC chromatogram as follows:

Mn, the numbered average molecular weight is calculated as follows:


$$Mn=\frac{\Sigma \left(Ni*Mi\right)}{\Sigma \;Ni}$$

where *i* is a slice (1.666 × 10^−3^ min) of the region, Mi is the molecular weight, and Ni is the intensity of the signal.

Mw, the weight average molecular weight, is calculated as follows:


$$Mw=\;\frac{\Sigma \;\left({Ni*Mi}^{2}\right)}{\Sigma \;\left(Ni\;*Mi\right)}$$

where *i* is a slice of the region, Mi is the molecular weight, and Ni is the intensity of the signal.

Polydispersity, PDI, is a measure of the broadness of the peak and is calculated as follows:


$$PDI=\frac{Mn}{Mw}$$

In this study, we focused on the polymeric parameters of region 1 of the chromatogram (70.5–1,020 KDa), i.e., the one excluding small-sized chitin oligomers (Mn_1_, Mw_1_, PDI_1_). The two other regions, namely region 2 (4.88–70.5 KDa) and region 3 (0.784–4.88 KDa) were not included in the analysis because (i) the chitin polymer subjected to incubation in sterile conditions in the salted culture medium produced COS eluting in region 2, and (ii) no significant differences were observed for the polymeric parameters of region 3 across the different samples.

Pearson correlations were computed between Mn_*1*_ and the height of settled chitin with the *ggscatter* function (*cor.method*= “pearson”) from the ggpubr package (v 0.5.0 (39);) in R. Correlations were also computed between the Mn_1_ and PDI_1_.

### Total community DNA extraction and 16S rRNA gene sequencing

DNA was directly extracted from 0.25 g of the inner sponge tissue, sediment, and from the seawater filter membranes for the environmental (*in situ*) samples and from the microbial cell pellet recovered from 5 mL of each enrichment culture (PC, C1, C2, and C3).

DNA was extracted using the DNeasy PowerSoil Pro kit from Qiagen (ID: 47014) according to the manufacturer’s protocol. The seawater filters were cut into small pieces using sterile scissors before DNA extraction. The DNA quantity (ng/µL) and quality (A260/A280 and A260/A230) were estimated with a NanoDrop ND 2000 UV-VIS spectrophotometer (Thermo Fisher Scientific, Waltham, US) and DNA samples were kept at −20°C until further analyses. 16S rRNA gene amplification and sequencing from DNA samples were carried out at StarSeq (Mainz, Germany) using Illumina MiSeq sequencing. The V4 region of the 16S rRNA gene was sequenced following a 2 × 300 paired-end approach using the primers 515F (5′-GTG YCA GCM GCC GCG GTAA-3′) and 806Rb (5′-GGA CTA CNV GGG TWT CTA AT-3′) (40, 41). An average of 100,000 paired-end sequences were generated per sample. The library of reads was demultiplexed by StarSeq.

### Processing of the 16S rRNA gene sequencing data

All 16S rRNA gene fragment sequences were primer-depleted and processed together using the denoising-based pipeline DADA2 (Divisive Amplicon Denoising Algorithm) v.1.8 (in R; (42)). First, the *FilterandTrim* function from DADA2 was used to remove the reads with Ns (*maxN =* 0). After combining all identical sequencing reads into “unique sequences” (each associated with the number of reads of each sequence) the DADA2 algorithm inferred amplicon sequence variants (ASVs). Paired-end sequences were merged (using the *mergeSequenceTables*() function from DADA2), and chimeric ASVs were removed (using the DADA2 function *removeBineraDenovo*() function) from the ASV table. Taxonomy (kingdom, phylum, class, order, family, and genus) of each ASV was then assigned using the SILVA database version 138.1 (43, 44).

The ASVs vs samples and taxonomy profile tables were then uploaded in R as a phyloseq object using the phyloseq package (v1.38.0; (45)) to perform diversity and taxonomic composition analyses. The data set was filtered using the function *subset_taxa* from the phyloseq R (v1.38.0 (45)) package to eliminate the mitochondria, chloroplast, eukaryote, and archaeal sequences. In the final data set (all sample types included), 2,505 bacterial ASVs were found (Table S6).

### Analysis of 16S rRNA gene fragments

#### Taxonomic composition

The final data set containing abundance distributions of bacterial ASVs across all samples was used to generate stacked bar charts in multiple combinations to facilitate data visualization. All barplots were generated using the R packages phyloseq (v1.38.0 (45)), dplyr (v1.8.6 (38)) and ggplot2 (v 3.4.0 (46)).

#### Alpha diversity

Alpha diversity metrics were determined for bacterial communities of environmental samples and enrichment cultures using non-rarefied data because all rarefaction curves reached a plateau, indicating that the bacterial diversity in each sample was exhausted with the sequencing depth employed here (Fig. S3). Observed bacterial richness (ASV counts) and diversity (Shannon–Wiener diversity index calculated from the abundance distributions of ASVs) were obtained for each sample using the *estimate_richness* function from the phyloseq package (v1.38.0 (45)).

For the bacterial communities of environmental samples, boxplots representing observed richness (ASV counts) and the Shannon index were plotted using the *qplot* function from the ggpubr package (v0.5.0; (39)). The normality and homoscedasticity of the data were confirmed using the Shapiro–Wilk and Levene tests, respectively, included in the car package (v3.1–1) in R (47). Then, an analysis of variance (ANOVA) test was performed to check whether differences in alpha diversity measures between groups of samples were significant. A *post hoc* Tukey test was then carried out to test for significant differences in alpha diversity measures between pairs of groups of samples. All statistical tests were performed in R version 4.1.2.

#### Beta diversity analyses

Two dedicated beta diversity analyses were performed in this study from the main data set: (i) one to determine whether differences in bacterial community structure occurred between environmental samples and (ii) one to determine whether such differences occurred among the different enrichment cultures (PC, C1, C2, and C3). For each analysis, the ASV data were first Hellinger-transformed (square root of ASV relative abundances). Then, a Bray–Curtis similarity matrix was calculated using the phyloseq package from R (v1.38.0 (45)). A principal coordinates analysis (PCoA) was generated for each analysis to ordinate the samples based on the Bray–Curtis matrix. Ordination diagrams were drawn using the ggplot2 package (v 3.4.0 (46)) in R.

To check for significant differences in bacterial community structure between biotopes and between enrichment cultures derived from biotope replicates, a permutational analysis of variance (PERMANOVA) was performed on each Bray–Curtis distance matrix with 999 permutations in R using the *adonis2* function from the vegan R package (v2.6–4 (48)). If the PERMANOVA test was significant, a *post hoc* test was performed using the function *pairwiseAdonis::pairwise.adonis* from the pairwiseAdonis R package (v0.4.1 (49)) to ascertain significant differences between pairs of groups. The PERMDISP test was conducted for each analysis using the *betadisp*() function from the vegan R package (48) to test for significant differences in dispersion between groups of samples.

For the analysis including only the environmental samples, ASVs that contributed most to community dissimilarities were identified with a similarity percentage (SIMPER) test on the Hellinger-transformed data using the PAST software (version 4.10 (50)). The 15 most differentiating ASVs were plotted in the PCoA graph.

### Assessing the coding potential for chitin degradation among the artificially selected bacteria

The IMG/M platform v7 (51) was used to search for pfam categories involved in chitin degradation among genomes of bacterial genera identified in this study as potential novel chitin degraders. On the IMG/M platform, we retrieved the number of available genomes for each of these genera and investigated the presence of sequences encoding domains of endochitinases [PF00182 (chitinase class I, GH19), PF00704 (GH18), PF08329 (chitinase A N-terminal domain), PF06483 (chitinase C)], exochitinases [PF03174 and PF13290 (Chitobiase/beta-hexosaminidase C-terminal domains), PF03173 (Chitobiase/beta-hexosaminidase N-terminal domain), PF02838 (GH20, domain 2), PF00728 (GH20, catalytic domain), PF14845 (beta-acetyl hexosaminidase like)], polysaccharide deacetylases [PF01522 (Polysaccharide deacetylase), PF04748 (Divergent polysaccharide deacetylase)], CBP [PF01607 (Chitin binding Peritrophin-A domain, family 14), PF02839 (Carbohydrate-binding module family 5/12)], and enzymes involved in N-acetylglucosamine utilization [PF01182 (Glucosamine-6-phosphate isomerases/6-phosphogluconolactonase)].

## RESULTS

### Bacterial community structure in environmental samples

**Fig 2:**
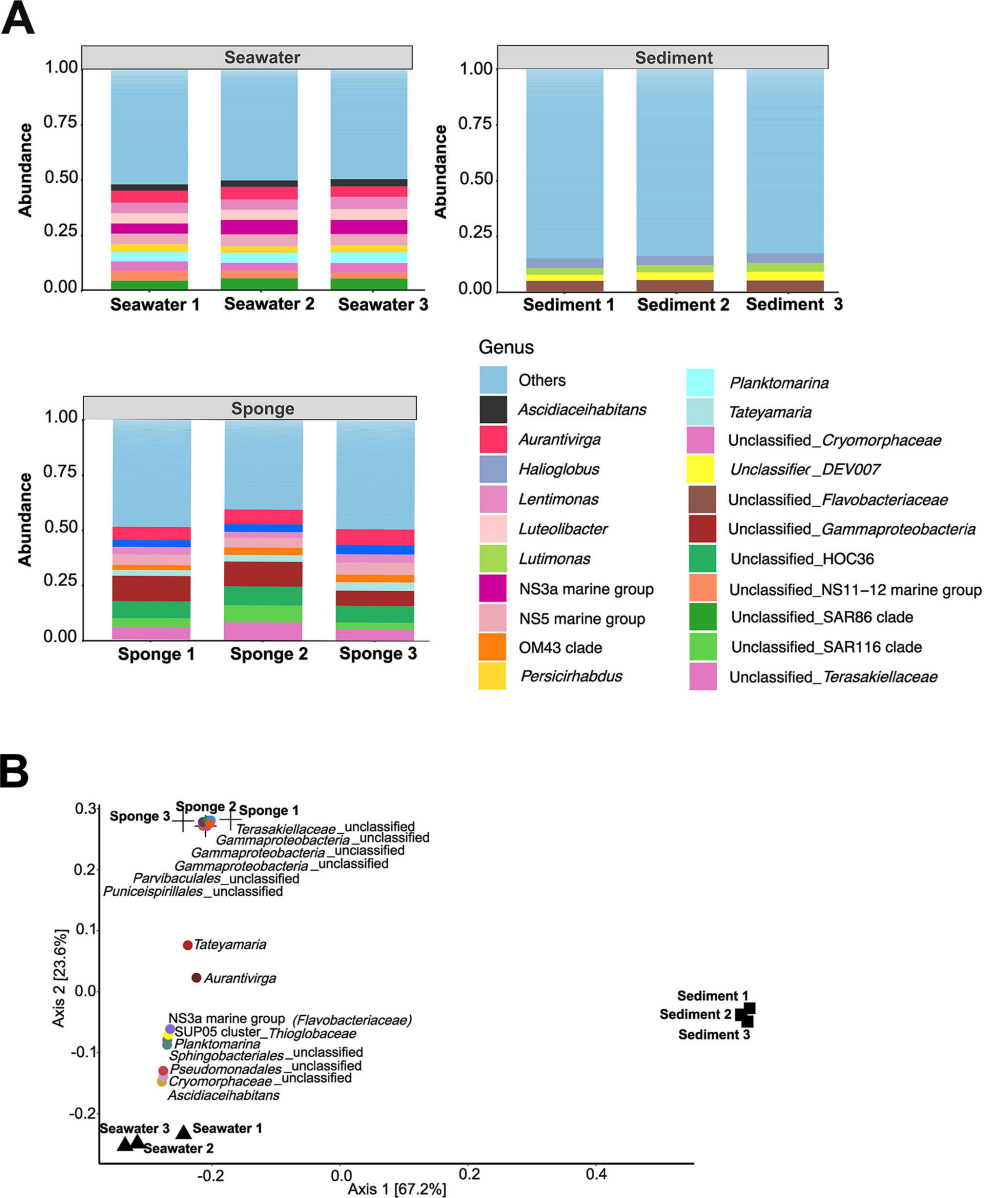
Bacterial communities in the three natural biotopes: the sponge *Hymeniacidon perlevis* and its surrounding seawater and sediment. (**A**) Taxonomic composition at the genus level. For each biotope, genera whose relative abundance were lower than 3% were merged into the category “Others.” (**B**) Principal coordinates analysis of seawater, sediment, and sponge bacterial communities. Community ordination was performed on a Bray–Curtis similarity matrix calculated after Hellinger transformation of the ASV relative abundances. Samples are represented by black shapes (sediment – triangles; seawater – circles; sponge – squares). The 15 ASVs that contribute the most to community dissimilarities among sample groups were plotted in colored dots. Their position in the ordination diagram reflects their relative abundance across all samples: the closer a phylotype to any given sample, the higher its relative abundance in that sample.

In the three environmental samples, the most abundant bacterial phyla were *Pseudomonata* (c. 30%, formerly *Proteobacteria*), *Bacteroidota* (c. 25%), and *Verrucomicrobiota* (c. 10%; Fig. S4A). However, sediment and sponge biotopes also hosted other abundant phyla: *Planctomycetota* (c.10%), *Desulfobacterota* (formerly *Desulfobacteria), Actinomycetota* (formerly *Actinobacteria*)*,* and *Chloroflexota* (formerly *Chloroflexi*; <5% each) in the sediment biotope, and *Cyanobacteria*, *Actinomycetota,* and *Planctomycetota* (<5% each) in the sponge biotope (Fig. S4A). At the family level, differences were observed between the three biotopes as well (Fig. S4B). At the genus level, low abundant taxa (i.e. genera with a relative abundance <3%) were less represented in the seawater and sponge samples (c. 50% of the genera) than in the sediment samples (c. 85% of the genera) (Fig. 2A). These results aligned with the significantly greater number of ASVs and alpha diversity observed in the sediment in comparison with the other biotopes (Fig. S5; Table S3). In the seawater samples, the most abundant genera, such as *Aurantivirga*, NS3a marine group (family *Flavobacteriaceae*), unclassified SAR86 (order *Pseudomonadales*), or *Planktomarina* all displayed around 5% of relative abundance (Fig. 2A). In the sediment samples, major taxa, such as *Halioglobus*, unclassified *Flavobacteriaceae*, *Lutimonas*, and unclassified DEV007 (order *Verrumicrobiales*), all represented around 5% relative abundance. Finally, majoritarian taxa of *H. perlevis* were unclassified *Gammaproteobacteria* (c. 11%), unclassified HOC36 (*Gammaproteobacteria*) (c. 8%), unclassified *Terasakiellaceae* (order *Rhodospirillales*) (c. 5%), and *Aurantivirga* (c. 6%).

The principal coordinates analysis (PCoA) performed on the ASV profiles of the bacterial communities of environmental samples showed a sharp separation of the three biotopes (Fig. 2B). This separation was statistically confirmed by a PERMANOVA test (*P* = 0.004) and not due to different dispersions of data points between the three biotopes (PERMDISP test, *P* = 0.09). The communities were significantly different between each pair of biotopes (pairwise adonis *P* = [0.002–0.01]). The biological replicates of each biotope clustered together, especially replicates of the sediment samples.

Among the 15 most differentiating ASVs (according to the SIMPER test), some ASVs were proximate to *H. perlevis* samples due to their higher relative abundance in the sponge samples (Fig. 2B). These were classified as *Parvibaculales* (ASV 171), *Puniceispiralles* (ASV 154), *Gammaproteobacteria* (ASVs 57, 161, and 265), and *Terasakiellaceae* (ASV 67). Some ASVs were located between the sponge and the seawater samples, which means that they were evenly represented in both biotopes while showing lower relative abundances in the sediment samples. These included ASVs classified as *Tateyamaria* (ASV 68), *Aurantivirga* (ASV 42), NS3a marine group (*Flavobacteriaceae;* ASV 70), SUP05 cluster (*Thioglobaceae;* ASV 252), *Planktomarina* (ASV 53), unclassified *Sphingobacteriales* (ASV 120), unclassified *Pseudomonadales* (ASV50), unclassified *Cryomorphaceae* (ASV 153), and *Ascidiaceihabitans* (ASV 82). Finally, none of the 15 most differentiating ASVs was proximate to the sediment group.

### Artificial selection experiments

The experimental setup for artificially selecting microbial communities is depicted in Fig. 1. Of the nine artificial selection experiments initiated (three replicates for each biotope), six were conducted until the final stage. Despite multiple attempts, one community from seawater (sample SW3) and two communities from sediment (samples SD2 and SD3) preserved in glycerol did not grow in the pre-culture medium according to the MTT assay.

#### Each enrichment culture developed differentially

**Fig 3:**
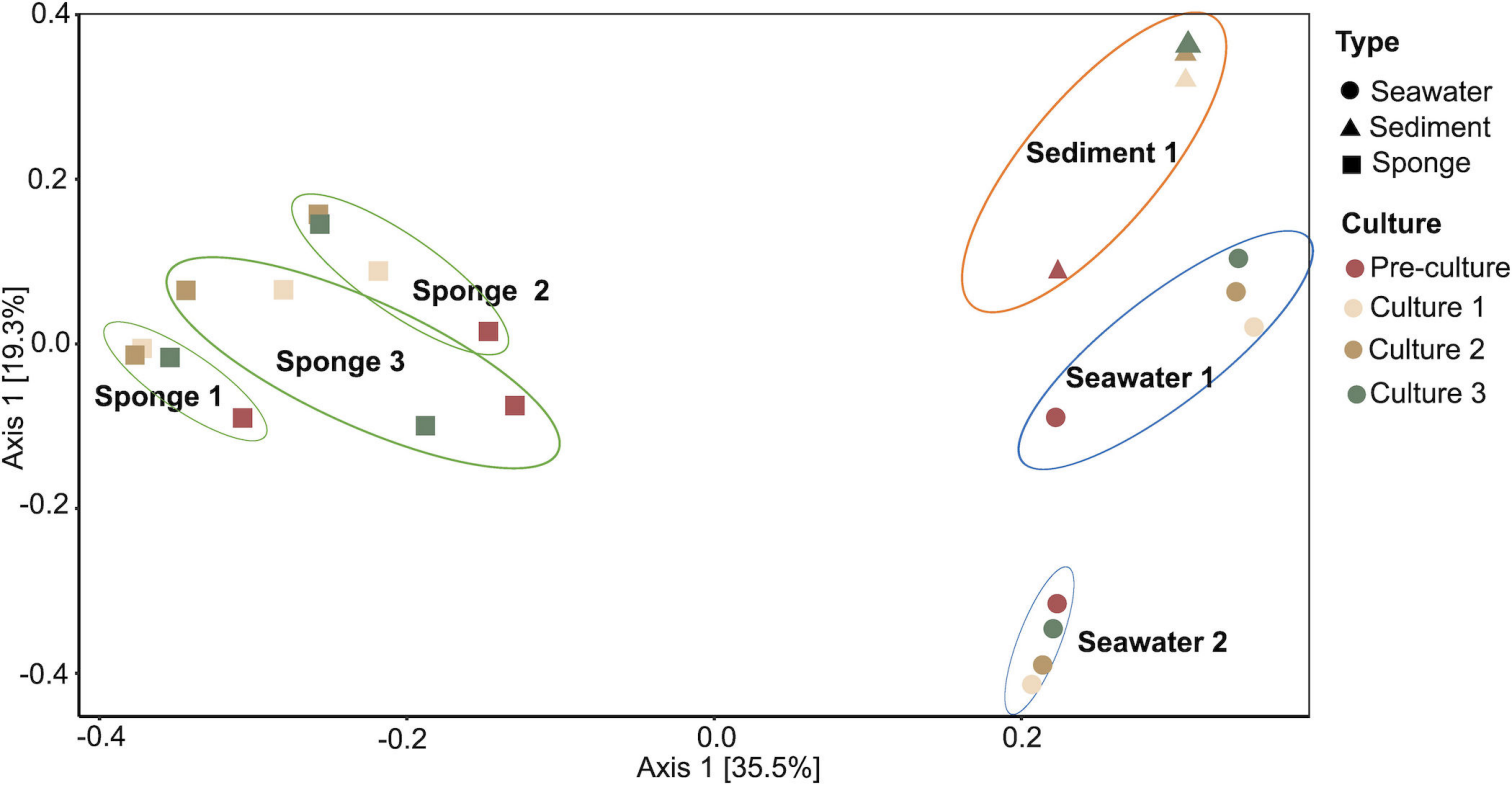
PcoA of bacterial communities from enrichment cultures performed on a Bray–Curtis distance matrix after Hellinger transformation of the ASV relative abundances. The different shapes represent the biotope from which the bacterial communities were obtained and used as first inoculum in the artificial selection experiment. The colors represent the enrichment steps (PC, C1, C2, and C3) during the artificial selection experiment.

The PCoA performed on all the enrichment cultures showed a clear separation of the cultures according to their source biotope and biological replicate (Fig. 3). This separation was statistically confirmed by a PERMANOVA test run on the six groups (i.e., SW1, SW2, SD1, SP1, SP2, and SP3) (PERMANOVA *P* = 0.001) and not due to differences in dispersion of data points between the six groups (PERMDISP *P* = 0.0989). Moreover, a pairwise ANOVA test confirmed that there was a significant separation between each pair of groups (*P* = [0.025–0.0037]), even for the replicates derived from the same biotope (i.e. SP1 vs SP2; SP1 vs SP3; SP2 vs SP3; SW1 vs SW2), which highlights the uniqueness of each enrichment event.

#### Sharp taxonomy shifts between environmental samples and enrichment cultures were marked by the selection of potentially novel chitin-degrading taxa

**Fig 4:**
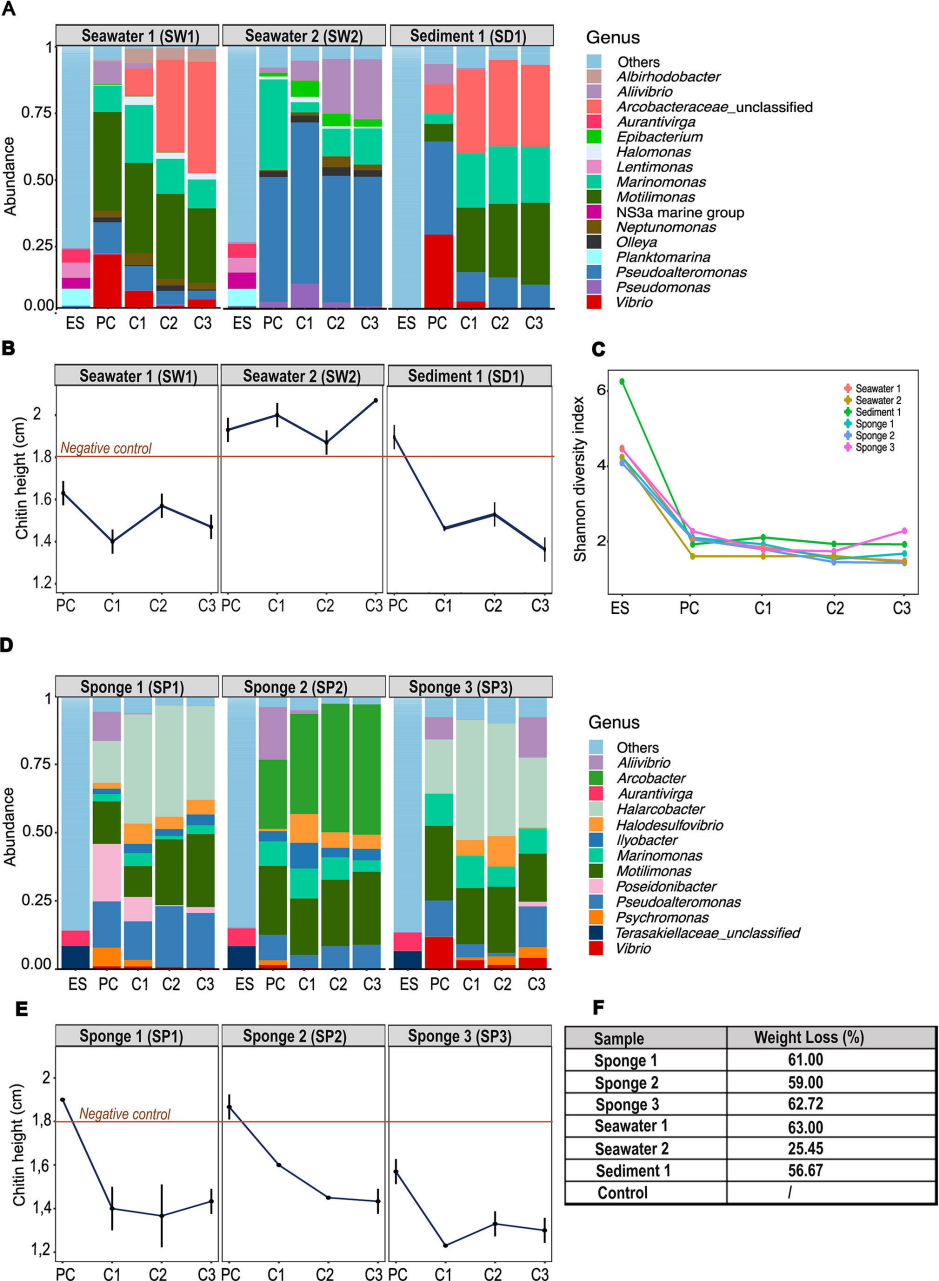
Taxonomic composition and Shannon diversity index of bacterial communities compared with measurements of chitin degradation during the artificial selection experiments. Genus-level taxonomic composition of bacterial communities in environmental samples (ES) and their corresponding enrichment cultures (PC, C1, C2, and C3) are presented for the three biotopes: seawater and sediment (panel A) and sponge (panel D). In each of the six subdatasets, genera whose relative abundance was below 1% were merged into the category “Others”. Plots displaying the height of the sedimented chitin (cm) are shown underneath the taxonomy bar charts (panels B and E). The chitin height of the negative control (chitin medium only) was 1.8 cm ± 0.1 cm. The Shannon diversity index of bacterial communities in environmental samples and their corresponding enrichment cultures is presented in panel C, and the percentage of chitin weight loss in cultures C2 is presented in panel F.

The strongest changes in taxonomic composition and diversity (Shannon index) were observed between the environmental samples and the enrichment culture PC whatever the source biotope replicate (Fig. 4A, C and D). Indeed, environmental samples exhibited a higher representation of genera with lower relative abundance (<1% across each data set) than the enrichment cultures (Fig. 4A and D). Besides, no strong changes in Shannon index were observed between PC and C1 cultures (although a clear shift in beta diversity was generally observed, Fig. 3) or between C1 and C2 cultures and between C2 and C3 cultures (Fig. 4C).

The dominant taxa in the enrichment cultures (*Pseudoalteromona*s, *Motilimonas, Marinomonas*, (*Hal)arcobacter*, unclassified *Arcobacteraceae*, and *Aliivibrio)* (Fig. 4A and D) were poorly represented (< 0,01%) in their corresponding environmental samples (Table S4), which underpins the large difference in taxonomic composition between the environmental samples and the enrichment cultures that emerged from the artificial selection process. Of note, the environmental samples also contained a variety of other genera reported to be chitinolytic in aquatic environments (17, 52): *Vibrio* (0.08-0.37% of the ASVs), *Shewanella* (0.03-0.05%), *Aquimarina* (0-0.03%), *Psychromonas* (0.03-0.27%), *Enterovibrio* (0-0.03%), and *Flavobacterium* (0-0.11%). Among those, *Vibrio* and *Psychromonas* were detected in the enrichment cultures at a relative abundance >1% (Fig. 4A and D). Finally, other chitinolytic genera were not detected at all in our environmental samples: *Serratia, Bacillus, Aeromonas, Microbulbifer, Enterobacter, Chromobacterium,* and *Erwinia*.

While *Marinomonas* (ASV7) and *Pseudoalteromonas* (ASV2) were identified in the enrichment cultures of all experiments, *Motilimonas* was totally absent from the enriched community where chitin degradation was low (SW2) (Fig. 4A, B, D and E; Table S4). Noticeably, different *Motilimonas* ASVs emerged in the enrichment cultures depending on the source biotope (e.g. ASV19 in SW1 experiment; ASV74+ASV19 in SD1 experiment and ASV3 in SP1, 2, and 3 experiments) (Table S4). Regarding the other genera, they were dominant in some enrichment cultures only. In the SW1 and SD1 experiments, unclassified *Arcobacteraceae* ASVs (ASV28 and ASV37 + ASV81 + ASV104, respectively) were dominant in the enrichment cultures C1, C2 and C3 (Fig. 4A; Table S4). In the SP1 and SP3 experiments, *Halarcobacter* (ASV5) was dominant in the enrichment cultures C1, C2, and C3, whereas in the SP2 experiment, it was *Arcobacter* (ASV4) (Fig. 4D; Table S4). Finally, in the SW2 experiment (the only one in which chitin degradation was low along the enrichment cultures), no *Arcobacteraceae* taxon was detected, whereas *Aliivibrio* (ASV8) emerged as one of the dominant taxa (Fig. 4A). The latter was present in the PC of the other experiments but disappeared in the subsequent enrichment cultures. In the four experiments, the genus *Vibrio* was present in the PC, but its relative abundance substantially decreased in the chitin-degrading cultures C1, C2, and C3.

Chitin degradation, as assessed by the chitin height measurement and chitin weight loss, was low along the SW2 experiment (Fig. 4B and F; Table S2). This result is in accordance with the low metabolic activity recorded for this sample by the MTT assay (Fig. S6). In the other experiments, enrichment cultures C1 and C2 were generally more efficient at degrading chitin than the enrichment culture PC. The strongest decrease in chitin height (and concomitant increase in cell activity) was indeed observed between PC and C1 enrichment cultures, after which chitin degradation generally stabilized. The lowest chitin height, corresponding to a chitin weight loss of 63%, was recorded for the SP3 enrichment cultures.

Interestingly, the relative abundance of some genera was correlated to chitin degradation, regardless of their ASV identity (with few exceptions). The relative abundance of all dominant ASVs affiliated with *Halarcobacter*, *Arcobacter*, and unclassified *Arcobacteraceae* was positively correlated to chitin degradation, except unclassified *Arcobacteraceae* ASV81 (Fig. S7). Dominant *Motilimonas* ASVs were either positively correlated to chitin degradation (ASV 19 and ASV 74) or not correlated but greatly represented (11% to 26%) during the whole enrichment culture process (ASV3). Conversely, all dominant ASVs affiliated with *Pseudoalteromonas*, *Vibrio*, *Aliivibrio*, *Psychromonas,* and *Poseidonibacter* were negatively correlated to chitin degradation (Fig. S7). The dominant ASV affiliated with *Marinomonas* (ASV7) was not correlated to chitin degradation (data not shown).

#### A shift in average molecular weight of the chitin polymer was observed in enrichment cultures where chitin was degraded

**Fig 5:**
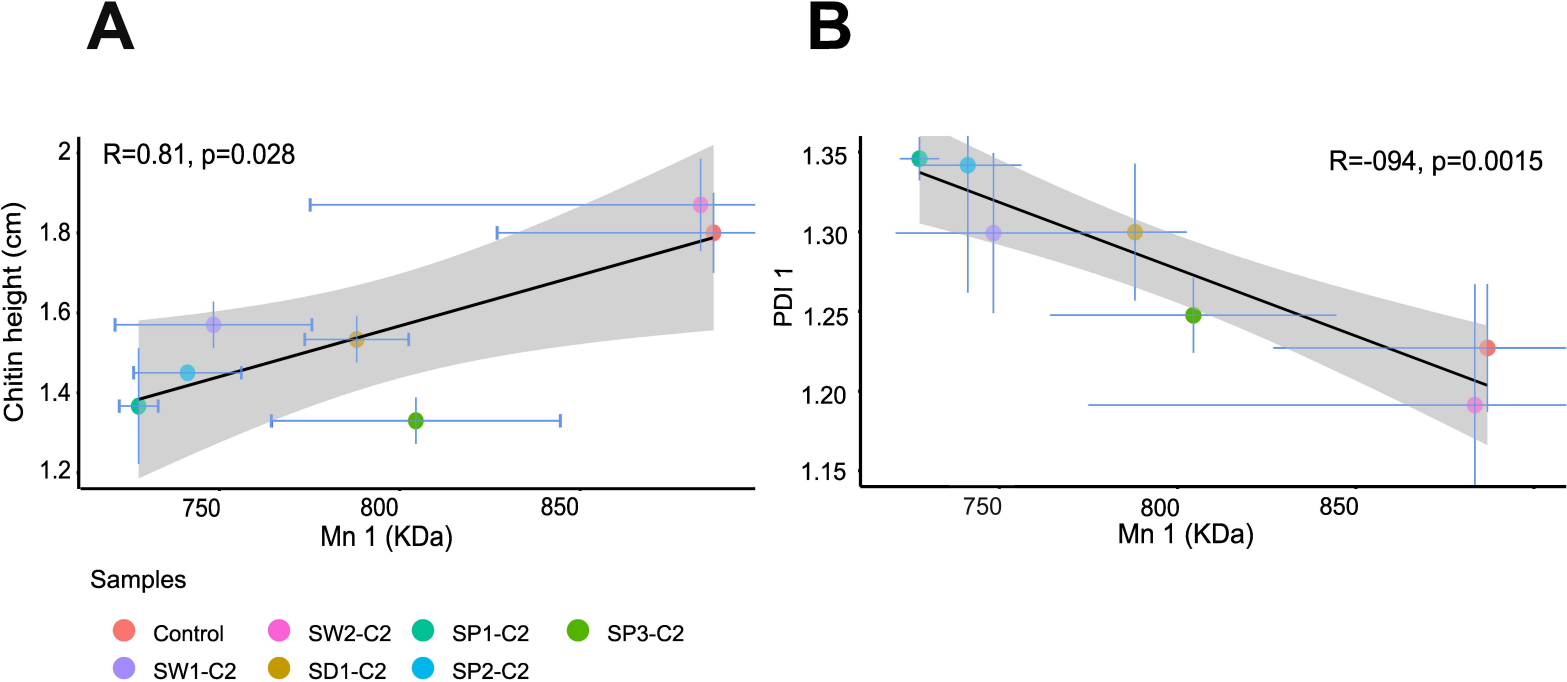
Correlations between parameters reflecting chitin degradation in the enrichment cultures C2. (**A**) Correlation between the numbered average molecular weight (Mn_1_) of the first region of the SEC chromatogram and the chitin height (cm). (**B**) Correlation between Mn_1_ and the polydispersity of the first region of the SEC chromatogram (PDI_1_). R and p values refer respectively to Pearson’s correlation coefficient and associated p-value. The light grey zone indicates the 95% confidence interval.

Mn_1_ and PDI_1_ values of all samples except SW2 were respectively smaller and higher than those of the negative control, highlighting a shift of the numbered average molecular weight of the chitin polymer in these experiments (Fig. 5A and B; Table S5, see Fig. S1B for example of shift in the chromatograms). Moreover, Mn_1_ values were significantly positively correlated with chitin height (Fig. 5A) and negatively correlated with PDI (Fig. 5B). In other words, communities degrading chitin best (low chitin height) degraded large-sized chitin polymers into smaller and more varied sizes (Mn_1_ decreased, PDI_1_ increased [Fig. S2]).

#### *Motilimonas* is likely a yet unidentified genus of chitin degraders

The genomes of strains belonging to the genera *Motilimonas, Arcobacter, Halarcobacter, Psychromonas, Marinomonas*, and *Poseidonibacter* available on IMG/M were screened for sequences encoding domains of proteins involved in chitin degradation (Table 1, detailed results in Table S7). Those genera were targeted because they were detected as dominant taxa in our experiments (Fig. 4) while they have been little or not reported in the context of chitin degradation in the literature so far. Sequences coding for domains of endochitinases were detected in the genome of all *Motilimonas,* 8% of *Arcobacter,* 28% of *Psychromonas,* and 2% of *Marinomonas* strains. Exochitinase sequences were detected in the genome of all *Motilimonas*, 2% of *Arcobacter*, and 28% of *Psychromonas*. Polysaccharide deacetylase sequences were detected in the genome of the great majority of strains belonging to the different genera. The same held true for N-acetylglucosamine utilization genes, except in *Arcobacter* strains where they were mostly absent. Finally, sequences coding for domains of chitin binding proteins were detected in the genome of all *Motilimonas* and 28% of *Psychromonas* strains. Compared with the other genomes, those of *Motilimonas* presented a considerably higher number of targeted sequences: 17 to 24 sequences per genome coding for domains of endochitinases, 3 to 4 for exochitinases , 5 to 11 for CBP and 3 to 4 for polysaccharide deacetylases (Table 1).

**TABLE 1:** Number of sequences coding for domains of endochitinases, exochitinases, carbohydrate-binding proteins (CBPs), polysaccharide deacetylases, and enzymes involved in N-acetylglucosamine utilization in the genomes available on the IMG/M system according to the pfam annotation^*a*^

Genus	*Motilimonas*	*Arcobacter*	*Halarcobacter*	*Psychromonas*	*Marinomonas*	*Poseidonibacter*
Number of genomes available	4	49	1	18	62	8
Endochitinases	20 (17–24)	0 (0–3)	0	4 (0–18)	0 (0–1)	0
Exochitinases	3	0 (0–1)	0	2 (0–10)	0	0
CBP	9 (5–11)	0	0	1 (0–3)	0	0
Polysaccharide deacetylases	4 (3, 4)	1 (0–2)	1	2 (1–4)	3 (2–5)	2 (1–3)
N-acetylglucosamine utilization	1	0 (0–1)	1	1 (0–2)	1 (0–1)	1 (0–1)

^*a*^ The numbers in parentheses correspond to the range of number of sequences retrieved accross the different genomes in each genus.

## DISCUSSION

In this study, we enriched microbial communities for (putatively novel) chitin degraders from three marine biotopes (a marine sponge and its surrounding seawater and sediment) of the same geographic location. The artificial selection process is a powerful technique to isolate consortia or pure strains involved in the biodegradation of compounds, such as xenobiotics, plant biomass, and other complex polymers (53–56). However, this process has only been applied in a few studies to recover chitin degraders from soils (57), estuaries (58), and seawater (55, 59). Recently, culture-independent metagenomic studies have evidenced that seawater, sediments, and sponges hold a great variety of chitinolytic microbes and their associated genes (17). This suggests that unique chitinolytic communities and distinct metabolic pathways dictate the processing of chitin across marine biotopes, and that artificial selection for chitin degrading consortia shall successfully expand from seawater (55, 59) to other underexplored marine niches.

### The *H. perlevis* bacterial community in the context of its environmental vicinities

Before enrichment cultures, we showed here that each natural biotope possesses its own unique bacterial community although the samples were collected in close proximity to each other (Fig. 2). Generally speaking, the microbiome of sediment and seawater differs from each other due to the different environmental conditions (i.e., pH, temperature, salinity, oxygen, and light) (60). Moreover, despite the fact that they continuously filter seawater, marine sponges are known to host sharply different microbiomes compared with those of seawater and sediments (61–63). In fact, sponges may acquire microbial symbionts by lateral acquisition (that is, via seawater filtering activity) as long as the symbionts are capable of evading the host’s digestive and immune systems (33, 64). Moreover, sponges can also acquire symbionts through vertical transmission (65). The bacterial community of *H. perlevis* specimens sampled at the intertidal zone of Bosham Harbour (UK) has been recently described, with emphasis on taxonomic composition at the phylum, order, class, and family levels (66). Here, we provide an in-depth depiction of bacterial communities associated with the same sponge from the French coast (beach of Audresselles) in light of the vicinal seawater and sediment communities. This allowed us to document the initial communities used in this study and compare them with those enriched during the artificial selection process. Low microbial abundance (LMA) sponges, the microbial abundance category to which *H. perlevis* most likely belongs, are less investigated than high microbial abundance (HMA) sponges (67). Our study identified the same dominant phyla (*Pseudomonata* [*Alphaproteobacteria* and *Gammaproteobacteria*] and *Bacteroidota*) and several identical families (*Flavobacteriaceae*, *Rhodobacteraceae,* and *Terasakiellaceae*) as the one conducted by Lamb and Watts (66) (Fig. S4). At the genus level, 10 dominant taxa accounted for about half of all ASVs, including *Aurantivirga* (order *Flavobacteriales*)*, Tateyamaria* (order *Rhodobacterales*), and *Lentimonas* (order *Opitutales, Verrucomicrobia)* (Fig. 2A). Among these, *Aurantivirga* and *Tateyamaria* were found to be shared with seawater samples (Fig. 2B), evidencing the contribution of the surrounding bacterioplankton to the diversity of the *H. perlevis* microbiome we captured. The co-occurrence of dominant bacterial species in seawater and LMA sponges is a well-documented trend (34), with such sponges usually sharing more bacterial species with their surrounding seawater than with their surrounding sediment (68). Concomitantly, we identified many other dominant taxa profusely enriched in the *H. perlevis* microbiome and absent from seawater. This was the case of the top three most abundant ASVs found in association with *H. perlevis*, namely ASVs 57 (unclassified *Gammaproteobacteria*), 67 (unclassified *Terasakiellaceae*), and 100 (unclassified HOC36 clade, class *Gammaproteobacteria*). Notably, these ASVs could not be classified at the order, family, and genus levels, respectively, highlighting the uniqueness of the *H. perlevis* microbiome. These specific symbionts may be recruited from the rare microbial biosphere of the surrounding environment or, alternatively, through vertical symbiont transmission processes.

### Discovery of new taxa potentially involved in chitin degradation through artificial selection experiments

Among the three biotopes that were sampled, the sponge *H. perlevis* was the only biotope from which we systematically selected chitin-degrading bacteria, as all enrichment experiments from that biotope succeeded. In addition, one sponge replicate (SP3) led to the most efficient consortium that was recovered in this study (Fig. 4).

In all six experiments where growth was observed in the preculture (PC), we observed a drastic shift in bacterial community composition, richness, and diversity between the environmental samples and the PC (Fig. 3 and 4). The cultivation process was responsible for this change (i.e., the procedure to isolate the cells, the conservation of samples, and the culture medium and cultivation conditions used in the enrichment cultures). It resulted in the consistent enrichment of taxa, such as *Marinomonas*, taxa from the *Arcobacteraceae* family and, particularly, *Motilimonas* from all sponge biological replicates, revealing that deterministic processes clearly shaped the enrichment of bacterial lineages from *H. perlevis*. However, in the same cultivation conditions, distinct enrichment cultures developed according to the specific biological replicate. Indeed, even if the three environmental samples of the sponge biotope were similar to each other (Fig. 2), stringent ordination analyses performed on ASV-level taxonomic profiles revealed that different enriched communities emerged from each biological replicate (Fig. 3). Stochastic processes may have introduced some degree of variability in the taxonomic composition of the enrichment cultures from the different experiments, for example the differential proportion of *Halarcobacter, Arcobacter*, or *Vibrio* ASVs across distinct sponge replicates as well as that of minor taxa.

In five of six experiments, chitin degradation improved over the successive enrichment cultures, usually at the C1 or C2 steps (Fig. 4). Our results revealed that the larger size chitin polymer was degraded into smaller size chitin polymers (Mn_1_ decreased, PDI_1_ increased) (Fig. 5; Fig. S2). This size change is likely due to the action of chitinases, especially endochitinases (69, 70). Indeed, this hypothesis is supported by the fact that several published genomes retrieved from online databases and corresponding to bacterial genera identified in our chitinolytic consortia possessed copies of endochitinase genes. This was the case of a few *Arcobacter, Psychromonas* and *Marinomonas* genomes and all *Motilimonas* genomes (Table 1). Moreover, other taxa that were dominant in our enrichment cultures, namely *Vibrio* and *Pseudoalteromonas,* are well known to be chitin degraders and to harbor endochitinase genes (12, 14, 17, 71–74). Finally, as the dominant taxa in the enrichment cultures were Gram-negative, chitin degradation might have been improved by the action of accessory, outer membrane-bound enzymes, such as the CBPs (detected repeatedly in the *Motilimonas* genomes) and LPMOs.

Comparing the efficiency of our enrichment cultures in degrading chitin with data from the literature is challenging as, to the best of our knowledge, only one study was based on raw chitin as the sole C and N source to isolate chitinolytic consortia (55). In addition, published studies dealing with chitinolytic consortia do not rely on remaining chitin height, remaining chitin weight or SEC to assess chitin degradation. A commercial chitinase assay kit containing monomers of short oligomers is commonly used instead (23, 55, 75). In another study, LC-MS was applied to quantify metabolites in the supernatant (76). However, the percentage of remaining mass of raw chitin was measured for several pure cultures of aquatic chitinolytic microbes. Depending of the strain and the culture conditions (temperature, agitation, etc.), the percentage of chitin degradation that was calculated ranged from 25% to 50% after 4–5 days (77, 78) to >90% after 30 days of incubation (78, 79). Considering the fact that we did not optimize the culture conditions nor the time of incubation to reach maximal degradation activity by our consortia but applied “mild” conditions instead (20°C, gentle agitation), we believe that 53%–67% of degradation of the initial chitin mass in 7 days is a promising result warranting future studies to optimize chitin conversion into COS and chitosan from symbiotic consortia.

Importantly, over the total duration of the experiments, we observed the maintenance of a mix of several taxa - the majority of which had been selected in the enrichment culture PC - and not one strain overtaking the others according to the competitive exclusion principle (80). The maintenance of diverse bacterial lineages during artificial selection most likely derives from the presence of various substrates resulting from chitin degradation in the medium, and by medium replenishment at the start of each enrichment step throughout the experiment. In a recent study performed in batches where the medium was not replenished, the interaction between a chitin degrader and a cross-feeder that consumed fermentation by-products was shown to evolve from mutually beneficial to competitive, highlighting the dynamic nature of such interactions over time (23). The co-existence of multiple taxa in our experiments suggests that chitin degradation was accomplished through the combined effort of taxa having different roles rather than by individual species, as it was repeatedly observed for recalcitrant substrates in the literature (55, 81–85). Yet, the precise role(s) of most dominant taxa in the enriched communities remain(s) to be discovered. Their contribution to chitin turnover can be hypothesized from the relationship between their relative abundance and chitin degradation across the different experiments and genomic data from closely related strains. *Arcobacter*, *Halarcobacter,* and unclassified *Arcobacteraceae* were prevalent in the final enrichment cultures C1, C2, and C3 where chitin degradation was >50% of the initial chitin mass and not represented in the final enrichment culture where chitin was less degraded (SW2 experiment) (Fig. 4). Plus, the majority of ASVs belonging to these taxa were positively correlated to chitin degradation across the different experiments (Fig. S7). Among the screened genomes of these taxa, a limited number harbored endochitinase and exochitinase sequences, and all of them had deacetylase sequences (Table 1), suggesting a potential activity of deacetylation of chitin and maybe of chitin hydrolysis. Likewise, *Poseidonibacter* (a genus closely related to *Arcobacter* (86)) and *Marinomonas* spp., whose relative abundance was not correlated to chitin degradation in our experiments (Fig. S7) but whose genomes harbored 1 to 5 deacetylase sequences (and no glycosyl hydrolases except one *Marinomonas* strain) (Table 1), could have survived as chitin-deacetylating members in the consortia. Reports on the involvement of these taxa in chitin degradation are lacking, and as far as we know, only one study mentions the ability of a *Marinomonas* strain to degrade colloidal chitin (87). Conversely, the relative abundance of *Motilimonas* ASVs was mostly positively correlated to chitin degradation (Fig. 4; Fig. S7), and the screened *Motilimonas* genomes were enriched in sequences coding for endo- and exo-chitinases, chitin-binding proteins, deacetylases, and enzymes involved in N-acetylglucosamine utilization (Table 1), suggesting a pivotal role of this taxon in chitinolytic consortia. One very recent study pointed to the presence of *Motilimonas* in the homogenate of a crushed Copepoda sample by 16S rRNA gene community sequencing (88). *Motilimonas* was not identified in the chitinolytic consortium enriched from that sample using a selective chitin-containing liquid medium, but it was identified in the enrichment culture obtained from a seawater sample using the same medium. These results align with our findings that *Motilimonas* can be detected in chitinolytic communities.

Finally, the different ASVs of *Vibrio*, *Aliivibrio*, *Pseudoalteromonas,* and *Psychromonas* were negatively correlated to chitin degradation in the different experiments (Fig. 4; Fig. S7). Such results were unexpected, as the chitinolytic activity of *Vibrio* and *Pseudoalteromonas* has been extensively reported and described in the literature. Likewise, it is demonstrated that the fish pathogen *Aliivibrio salmonicida* can degrade and metabolize chitin (89). Finally, although studies on *Psychromonas* are scarce, the ability of one *Psychromonas* isolate to readily degrade chitin hydrogel was described (90, 91), and several *Psychromonas* genomes that we screened contained sequences of proteins involved in chitin binding, hydrolysis, and deacetylation (Table 1). Our results suggest that under the conditions of the experiments, these taxa were outcompeted by better chitin degraders and/or suboptimally acclimated to the medium and incubation conditions. Furthermore, considering that the chitin used in this study was α-chitin, which is particularly challenging to break down due to its robust hydrogen bond (92), it is possible that these strains struggled to degrade it. For example, one study has reported that *Vibrio harveyi* grows better and expresses more chitinase activity in a liquid medium with β-chitin than with α-chitin (71). Interestingly, in a previous study that used the microbial community from bulk marine debris as inoculum for the artificial selection of chitin-degrading consortia, *Vibrio* was not recovered in high relative abundance either (c.2-3%) (55). Likewise, expected chitin degraders *Pseudoalteromonas* and *Aliivibrio* were dominant in the sole experiment where chitin degradation was low (SW2) (Fig. 4A), suggesting that these taxa may be poor chitin degraders in the experimental conditions of this study.

### Conclusion and perspectives

In this study, we brought novel insights into the microbiome of the marine sponge *H. perlevis*. Indeed, we showed that the microbiome of *H. perlevis* shares several genera with the surrounding seawater column. Next, we succeeded at selecting bacterial consortia that degrade efficiently raw chitin and discovered community members likely to play a pivotal role in chitin hydrolysis and deacetylation, such as *Motilimonas* and taxa belonging to the *Arcobacteraceae* family (e.g. *Arcobacter*, *Halarcobacter*). Moreover, our data demonstrate that applying the artificial selection process to a range of distinct marine biotopes increases the discoverability of novel chitin-degrading taxa and consortia, as different ASVs from *Motilimonas* and from the *Arcobacteraceae* family were retrieved. The recovery of chitinolytic consortia with microbes unknown for their catalytic potential towards chitin opens new ground to the future discovery and characterization of novel enzymes of marine origin involved in chitin degradation processes. The ongoing research effort in our laboratory, focusing on metagenomic and metatranscriptomic analyses of the selected communities and the isolation of strains from the consortia, will contribute to a better understanding of the function(s) of the different members in the chitin-degrading consortia, and how different environmental microbes balance their multiple enzymatic activities over time during the complex process of chitin degradation.

## Data Availability

The amplicon sequencing data (Table S8) are available in the Sequence Read Archive (SRA) under the project accession number PRJNA953361, sample accession numbers SAMN34111365 to SAMN34111371, SAMN34111833 to SAMN34111844 and SAMN34112193 to SAMN34112212; run accession numbers from SRR24102364 to SRR24102370, SRR24105266 to SRR24105277 and SRR24105279 to SRR24105298.

## SUPPLEMENTAL MATERIAL

Supplemental figures spectrum.00886-24-s0001.pdf Supplemental methods; Fig. S1 to S7.

Supplemental tables spectrum.00886-24-s0002.xlsx Tables S1 to S8.

ASM does not own the copyrights to Supplemental Material that may be linked to, or accessed through, an article. The authors have granted ASM a non-exclusive, world-wide license to publish the Supplemental Material files. Please contact the corresponding author directly for reuse.
