# Supplementary material for: Selection of marine bacterial consortia efficient at degrading chitin leads to the discovery of new potential chitin degraders
Source: Microbiol Spectr. 2024 Sep 24;12(11):e00886-24. doi: 10.1128/spectrum.00886-24 (PMC11537107; doi:10.1128/spectrum.00886-24)
Supplement: Supplemental figures — Supplemental methods; Fig. S1 to S7. [file spectrum.00886-24-s0001.pdf]

Supplementary material

Methods

MTT viability assays

This assay was adapted from (Chang et al., 2011). Briefly, 60 µL of MTT (tetrazolium salt; Sigma-Aldrich/Merck, Germany) were added to 200 µL of each enrichment culture (in technical triplicates) in a sterile 96-well microplate. Microplates were incubated for 30 min at 37 °C. Then, plates were centrifuged at 10,000 g for 7 min and the supernatant was removed. The remaining, reduced formazan cell pellet was dissolved in 200 µL of pure DMSO, the microplates were centrifuged again at 10,000 g for 7 min, and the absorbance of the supernatant was measured at 570 nm (A570) . If the A570 value of a sample was higher than 0.3 it was interpreted as an indication of bacterial growth. The A570 value of the negative control -chitin-based enrichment culture medium incubated under the same conditions as the (pre)cultures but without bacteria - was 0.04.

Size exclusion chromatography protocol:

The dry pellet of cultures C2 (5 mL) was solubilized into chitosan. To deacetylate the chitin polymers, 1 mL of NaOH 50% was added to 10 mg of chitin (adapted from (Min et al., 2004; Galed et al., 2008; Teng D., 2011)). The reaction tubes were vortexed for 5 sec and then incubated at 60 °C for 1 day. Afterwards, the obtained chitosan was centrifuged (5 min at 7000 rpm) and the chitosan pellet washed three times with MilliQ water. SEC was performed at ≈ 25°C using a combination of one pre-column PSS 10µ NOVEMA 3000 Å (8 mm diameter x 50 mm length; Polymer Standards Service GmhH, Mainz, Germany) and three columns 10µ NOVEMA 3000 Å NOVEMA MAX high (8 mm diameter x 300 mm length; Polymer Standards Service GmhH, Mainz, Germany) connected sequentially using an UHPLC system (Dionex Ultimate 3000, Sunnyvale) equipped with a refracting index sensor. The eluent, an aqueous solution of formic acid 0.03% and $NaNO_3$ 0.05 M, was pumped in isocratic mode at a flow rate of 1 mL/ min. Injected samples contained all 1.5 mg of chitosan (processed upstream from chitin) dissolved in 1 mL of eluent. Linear pullulan with a range from 0.180 to 1020 KDa (ReadyCal-Kit Pullulan high from Polymer Standards Service GmhH, Mainz, Germany; Fig. S1A) was used as standard to calculate the regression equation to convert the obtained retention

time into molecular weight of the chitosan polymer (which results from the conversion of chitin).

Figures

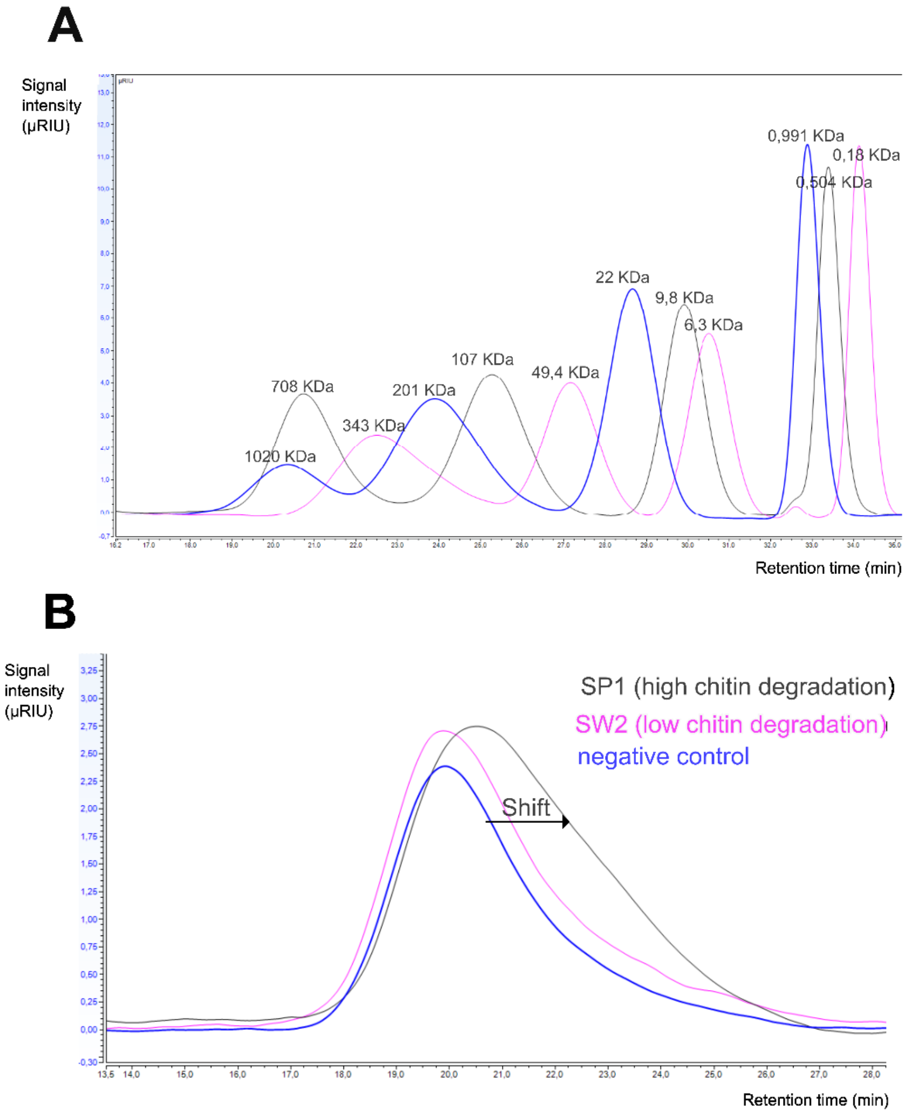

Figure S1 : SEC chromatograms computed by Chromeleon software. A) represents the standards of linear pullulans with the molecular weight of each peak indicated graphically, those were used to calculate the molecular parameters of the unknown samples starting from the retention time and refractive index RI signal, B) represents the main region of the obtained chitin chromatogram (including molecular weights from 80 KDa to >1.000 KDa), defined as region 1. We report 3 significant cases: a native undigested chitin or negative control (blue line); a sample in which the chitin was extensively degraded (SP1 black line; Mn_1 decreased

compared to Mn_1 the control); finally a sample in which chitin was marginally degraded (SW2; pink lines; Mn_1 is similar to Mn_1 the control).

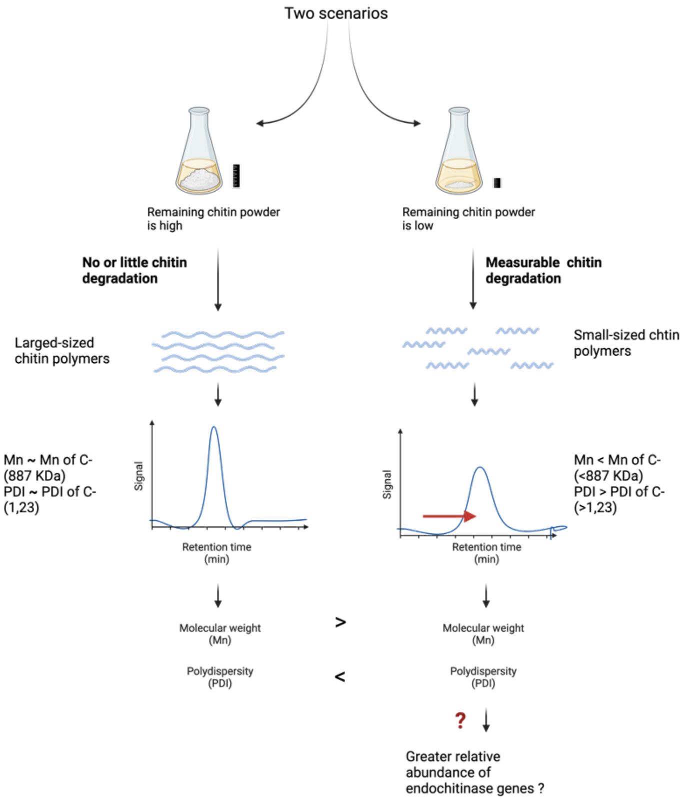

Figure S2 : Characterization of the chitin degradation products when chitin was not (or marginally) degraded and when it was well degraded by the bacterial consortia. Image created with BioRender.com

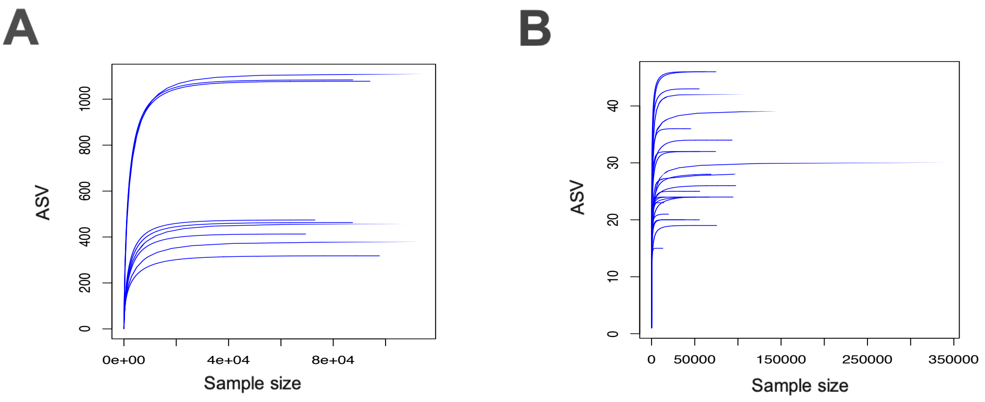

Figure S3 : Rarefaction curves showing the number of ASVs vs the number of reads in environmental samples (A) and in enrichment cultures (B).

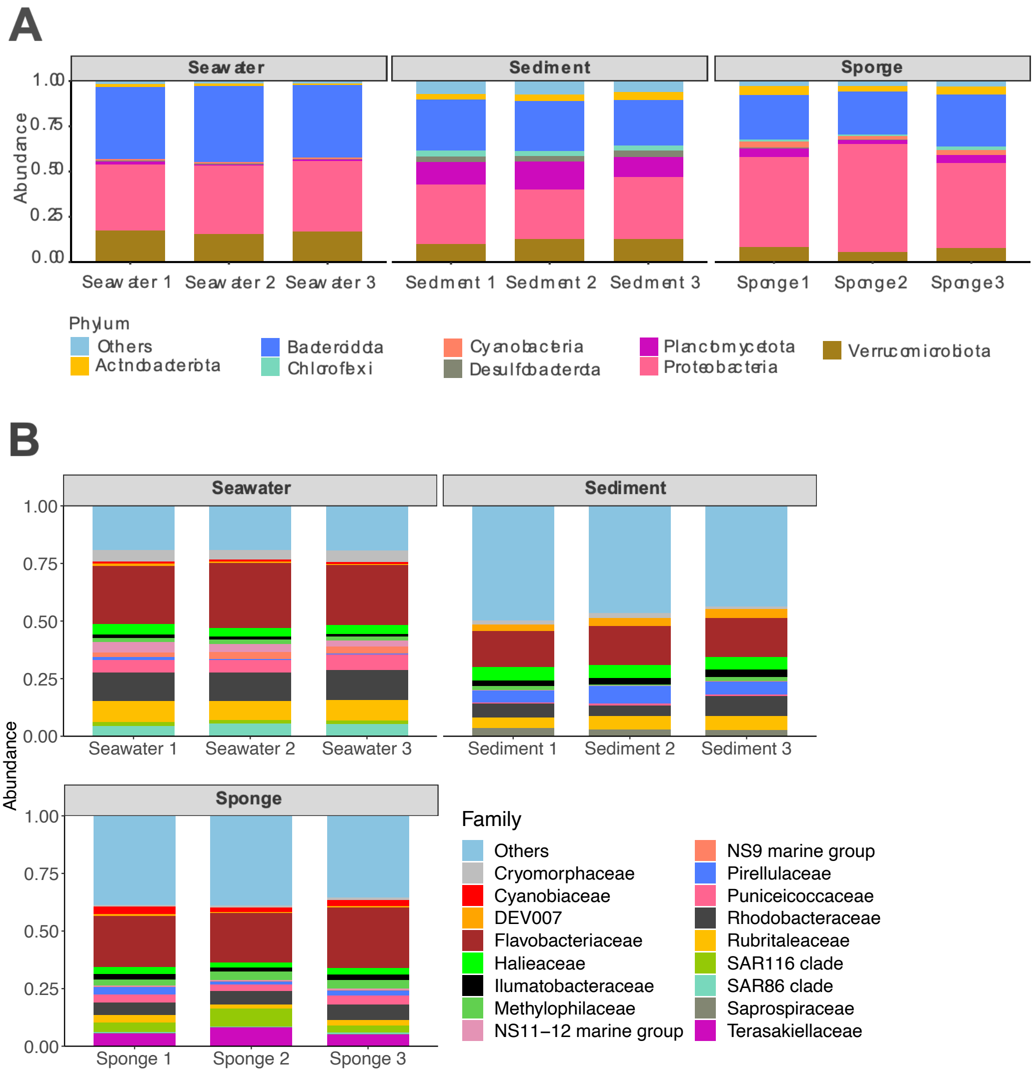

Figure S4 : Taxonomic composition at the Phylum level (A) and at the Family level (B) of the bacterial communities of the three natural biotopes (in biological triplicates). For each biotope, phylum and family whose relative abundance was below 1 % were merged into the category "Others" (light blue labelled bars).

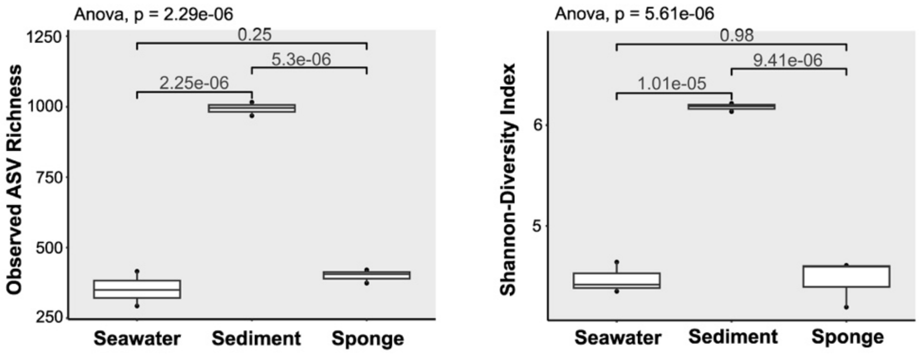

Figure S5 : Alpha diversity (Observed richness and Shannon-Wiener diversity index at the ASV level) of the bacterial communities in the three natural biotopes (in biological triplicates). Boxes represent the interquartile range (IQR) between the first and third quartiles (25th and 75th percentiles, respectively), and the horizontal line inside the box defines the median. Observed richness and Shannon diversity index were significantly different across biotopes (Anova p-value < 0,05). The P-value of significant pairwise differences between biotopes (Tukey test p-values < 0,5) is reported over the boxplots.

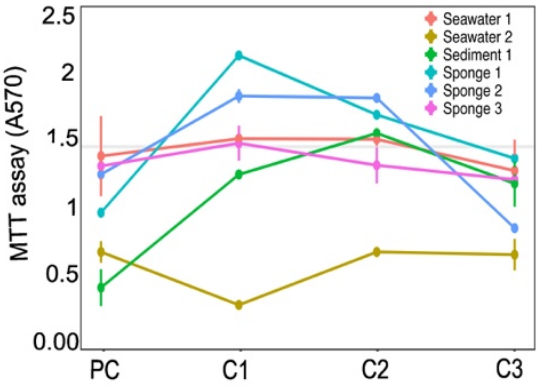

Figure S6: General metabolic activity during the artificial selection experiments measured by the MTT assay (Absorbance at 570 nm).

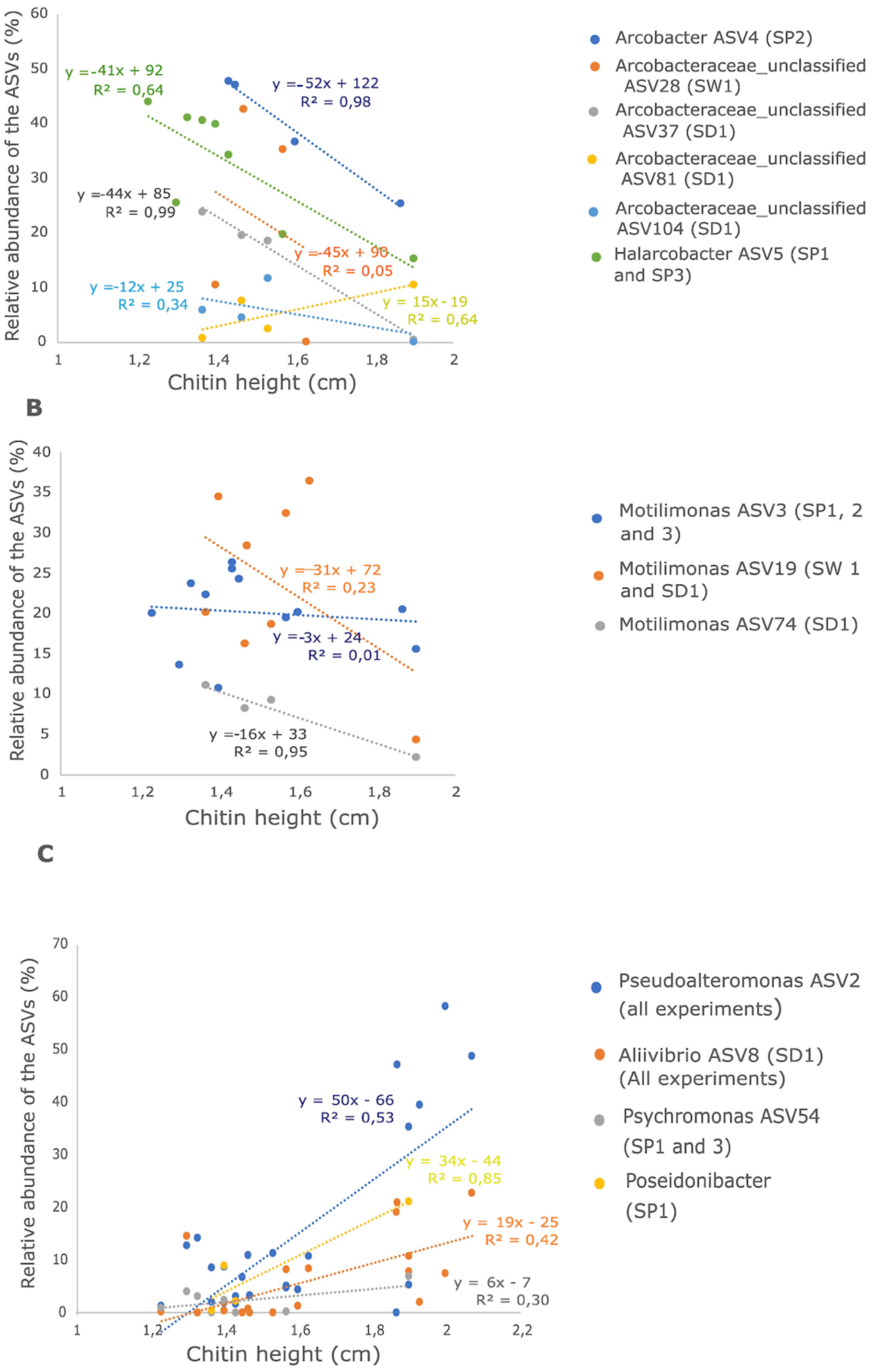

Figure S7 : Relative abundance (in %) of the dominant ASVs vs chitin height (cm). A dominant ASV in a selection experiment was defined as an ASV representing at least 5% of the community in at least one of the enrichment cultures of that experiment. Chitin height is inversely proportional to chitin degradation.
